# Optimizing Bone Health in Duchenne Muscular Dystrophy

**DOI:** 10.1155/2015/928385

**Published:** 2015-06-01

**Authors:** Jason L. Buckner, Sasigarn A. Bowden, John D. Mahan

**Affiliations:** ^1^Division of Endocrinology, Department of Pediatrics, Nationwide Children's Hospital, The Ohio State University College of Medicine, Columbus, OH 43205, USA; ^2^Division of Nephrology, Department of Pediatrics, Nationwide Children's Hospital, The Ohio State University College of Medicine, Columbus, OH 43205, USA

## Abstract

Duchenne muscular dystrophy (DMD) is an X-linked recessive disorder characterized by progressive muscle weakness, with eventual loss of ambulation and premature death. The approved therapy with corticosteroids improves muscle strength, prolongs ambulation, and maintains pulmonary function. However, the osteoporotic impact of chronic corticosteroid use further impairs the underlying reduced bone mass seen in DMD, leading to increased fragility fractures of long bones and vertebrae. These serious sequelae adversely affect quality of life and can impact survival. The current clinical issues relating to bone health and bone health screening methods in DMD are presented in this review. Diagnostic studies, including biochemical markers of bone turnover and bone mineral density by dual energy X-ray absorptiometry (DXA), as well as spinal imaging using densitometric lateral spinal imaging, and treatment to optimize bone health in patients with DMD are discussed. Treatment with bisphosphonates offers a method to increase bone mass in these children; oral and intravenous bisphosphonates have been used successfully although treatment is typically reserved for children with fractures and/or bone pain with low bone mass by DXA.

## 1. Introduction

Duchenne muscular dystrophy (DMD) is an X-linked recessive disorder (one of the dystrophinopathies) that is due to mutations in the dystrophin gene, encoding the dystrophin protein. This mutation leads to a deficiency of dystrophin, a protein that stabilizes the extracellular matrix and the cytoskeleton. Cell membrane destabilization, via loss of dystrophin, results in necrosis of myofibers and progressive muscle weakness [1]. Affecting approximately 1 in 3,500–5,000 males, it is the most common and severe form of muscular dystrophy [2]. DMD is usually marked by onset of symptoms between the ages of 3 and 5 years. The early symptoms are typically related to gait, including delayed onset of walking, toe walking, and/or a waddling gait. Serum creatine kinase (CK) is typically elevated 50 to 100 times above normal. Historically, loss of ambulation occurred between the ages of 7 and 12 years and death by the end of the second decade. In nearly 90% of cases, DMD is due to mutations that truncate the reading frame, leading to an absence of dystrophin expression.

In patients with DMD, prior to corticosteroid treatment, muscle weakness would inevitably progress and early in the second decade of life these boys would become nonambulatory and eventually require respiratory support. Two corticosteroids commonly used to treat DMD are prednisone and deflazacort, an oxazolone derivative of prednisolone [3–5]. Five recently published, long-term controlled nonrandomized trials (now extending beyond 3 years) with prednisone or deflazacort [6] demonstrated that, with either drug, patients retain muscle function longer, ambulate 2 to 5 years longer [4, 5], require less spinal stabilization surgery [7], and have delayed need for noninvasive ventilation [8–10] and less cardiac dysfunction than boys not receiving corticosteroid therapy [10–12].

Corticosteroid therapy along with better supportive care for cardiopulmonary status has improved survival in DMD from an average of 14.4 years in the 1960s to an average of 24.7 years according to a report published in 2010 [6]. The beneficial effects of corticosteroids in DMD are thought to be due to their potent anti-inflammatory activity that reduces the inflammatory response in dystrophin-deficient muscle, which in turn delays the loss of muscle strength and preserves the ability to ambulate longer than in boys without corticosteroid therapy. Unfortunately, this therapy comes with many adverse effects including negative effects on bone health such as low bone mass and bone fragility fractures [13–19]. For a more detailed discussion of corticosteroid therapy concerning timing of initiation, treatment after loss of ambulation, as well as the mechanism of corticosteroids, and other treatments in DMD, the reader is referred to recent reviews [20, 21]. In this review, we present the current risks of poor bone health, screening methods, diagnostic studies, and treatments to optimize bone health in patients with DMD.

## 2. Risks to Bone Health

Several pathophysiological mechanisms can contribute to the deterioration of bone health seen in DMD patients. Mechanisms that can alter bone health in children with DMD are as follows:

1. Progressive muscle weakness.
2. Effects of cytokines released due to the inflammatory response in dystrophin-deficient muscles.
3. Activation of osteoclastogenesis by altered muscle metabolism.
4. Corticosteroid side effects:
  - delayed puberty with decreased sex steroids,
  - impaired osteoblast bone formation/mineralization,
  - poor calcium absorption from the intestine.
5. Immobilization effects on bone calcium homeostasis.
6. Insufficient vitamin D stores:
  - poor vitamin D absorption from corticosteroids,
  - reduced sun exposure from lack of outdoor physical activity.

Many patients experience multiple factors and corticosteroid therapy is associated with additional risk factors for poor bone health. Genetic factors that may contribute to poor bone mineralization or exaggerated bone mineral loss have not been explored.

## 3. Bone Fragility in DMD

King et al. found that long bone fractures were 2.6 times more frequent in DMD patients treated with steroids than steroid naïve patients [16]. The steroid naïve group also had no vertebral fractures while 32% of the steroid treated group had vertebral fractures [16]. About 20–25% of DMD patients have a long bone fracture [22], and those of the lower extremities often result in the onset of permanent loss of ambulation [23]. Immobilization, such as full-time wheelchair use, can further alter bone health and the risk for fracture by causing additional demineralization of bone [24, 25].

Tian et al., in a recent retrospective study in a large cohort of 408 glucocorticoid-treated DMD patients aged 3–19 years, demonstrated that fracture prevalence increased progressively with age, along with worsening motor function [26]. Prevalence of total fractures was 16.5%, 37.4%, and 83.3% at ages 5, 10, and 18 years, respectively. Prevalence of vertebral compression fractures was 4.4%, 19.1%, and 58.3% at the same ages. This high prevalence of fractures with increasing age heightens the importance of monitoring bone health in DMD patients and providing interventions to improve bone health in this susceptible population [26].

## 4. Bone Mineral Density (BMD) in DMD 

In 2000, Larson and Henderson [23] described bone density findings in 36 corticosteroid naïve DMD boys. They showed that BMD was decreased in boys with DMD compared to normal controls but also that the mean lumbar spine *Z*-score of 17 ambulatory DMD boys (−0.8 ± 0.2) decreased further with the loss of ambulation (mean lumbar spine *Z*-score −1.7 ± 0.2) (*P* = 0.004). In a group of 32 DMD boys (22 on prednisone and 10 corticosteroid naïve), Bianchi et al. [17] found that cumulative corticosteroid dose correlated to decreased BMD (*P* < 0.05) and better lower limb muscle strength correlated with better BMD (*P* < 0.01). They also showed that BMD was lower in DMD boys than in normal control patients at the spine and total body, but no significant difference was seen in the spine *Z*-scores once adjusted for body surface area or for vertebral volume. Mayo et al. [27] followed 39 DMD boys on deflazacort therapy for about 8 years and demonstrated that there was a decrease in the age-adjusted BMD *Z*-score within the first 2 years of starting deflazacort (*P* < 0.05), with a consistent decline thereafter; in those who lost ambulation, the decline was greater. Compared to age-based *Z*-scores, height-adjusted BMD *Z*-scores were less impaired in this population. This may reflect better bone health than what is anticipated in these DMD boys on corticosteroids; since children on corticosteroids tend to be short, when their *Z*-scores were corrected for their smaller size, their BMD was not as poor as it would be when compared to normal heighted control boys.

## 5. Bone Health Screening

Leung et al. reported screening recommendations derived from a 2010 DMD conference [28]. Suggested screening included (1) nutritional and fracture history, (2) reviewing radiographic evidence of past fractures, (3) obtaining a back pain assessment on initial and each subsequent visit, and (4) checking the serum 25-hydroxyvitamin D level every 1-2 years prior to steroid therapy and maintaining a consistent level ≥30 ng/mL (≥75 nmol/L). Upon starting steroids, a dual energy X-ray absorptiometry (DXA) scan of the lumbar spine and a lateral spinal radiograph is recommended to screen for low BMD and vertebral fractures, respectively. Once a DMD patient has symptoms/signs of vertebral fracture, starting bisphosphonate therapy is recommended; in regard to bisphosphonate treatment before vertebral fractures, this should be decided on a case by case basis.

Similar screening suggestions were derived from a 2009 DMD conference as summarized by Quinlivan et al. [29]. Major additional recommendations included (1) encouraging physical activity and standing and questions related to sun exposure (as a source of vitamin D) at initial and subsequent visits, (2) performing a baseline DXA, and (3) repeating the DXA every 12–24 months if the body size corrected lumbar spine *Z*-score is >−2 and no back pain is present. If the lumbar spine *Z*-score is <−2 or there is back pain, then lateral imaging is required for vertebral fracture screening. Once a vertebral fracture is present, bisphosphonate treatment should be considered.  Table 1 outlines a summary of the bone health screening and course of action recommendations for boys with DMD [28, 29].

As studies have suggested, mobility influences bone density [30] and fracture risk [24]. There are several different mobility scales used in DMD to measure muscle function. Some scales are based on timed functional tests, such as the 6-minute walk test, 2-minute walk test, and time to rise from the floor (lying supine on the floor to full standing position). Others are based on clinical evaluation, such as the Vignos lower extremity functional grade and Brooke upper extremity functional grade [31–33]. To our knowledge, there are no reported studies that correlate mobility scales to BMD and fracture risk.

### 5.1. Biochemical Screening

#### 5.1.1. Calcium and Vitamin D

Screening for calcium intake can be completed by asking patients about the amounts, types, and frequency of dairy products they consume. Vitamin D intake can also be assessed from the diet history or 72-hour dietary record. It is more difficult to quantify and qualify the amount of sun light a child obtains. However, a simple blood test (serum 25-hydroxyvitamin D level) can screen for vitamin D deficiency. The Endocrine Society defines 25-hydroxyvitamin D levels of <20 ng/mL as deficient, 20–30 ng/mL as insufficient, and ≥30 ng/mL as sufficient [34].

#### 5.1.2. Bone Formation and Turnover Markers

Markers for bone formation and turnover have been examined in a limited number of DMD patients. These include bone formation markers, such as osteocalcin (OC) and bone-specific alkaline phosphatase (BALP), and bone breakdown products, such as pyridinoline, deoxypyridinoline, type 1 procollagen intact amino-terminal propeptide (PINP), carboxyterminal telopeptide of type 1 collagen (ICTP), N-terminal cross-linked telopeptide (NTx), and osteoclast-derived, tartrate-resistant, acid phosphatase isoform 5b (TRACP-5b) [17, 18]. Research shows that the values for these markers change with age, but currently there are no well-established normal ranges for different ages for some of the newer markers [35]. Bisphosphonates have been shown to decrease bone turnover markers (BALP, OC, NTx, and pyridinoline) in other patient populations [36]. One study compared OC and NTx in DMD patients treated with and without corticosteroids to a normal control group [17]. They found that while the OC was in the upper limit of normal, the NTx was nearly 4 times higher than the control group. An additional study showed that, for DMD patients on corticosteroid treatment, BALP, PINP, OC, CTX, and TRACP-5b were decreased compared to controls [18]. Clearly, further research is needed to evaluate the clinical utility of bone turnover markers in assessing bone fragility, predicting fractures, and monitoring treatment in this population. Until bone turnover markers are better understood, they cannot be recommended for bone health screening, at least at this time [29].

### 5.2. Imaging Screening

#### 5.2.1. Spinal Assessment

Chronic glucocorticoid use has a significant impact on trabecular bone, particularly vertebra, which can result in vertebral compression fractures. The prevalence of symptomatic vertebral fractures in corticosteroid-treated DMD ranges from 32 to 40% [14, 16]. The frequency of asymptomatic vertebral fractures DMD is unknown. It has been shown that 10% of patients with juvenile idiopathic arthritis on glucocorticoids had asymptomatic vertebral fractures [37]. More generally, vertebral fractures are often asymptomatic in children with low bone mass with only 18%–56% of diagnosed vertebral fractures in this population presenting with clinical signs and symptoms in children [38, 39].

**Figure 1:**
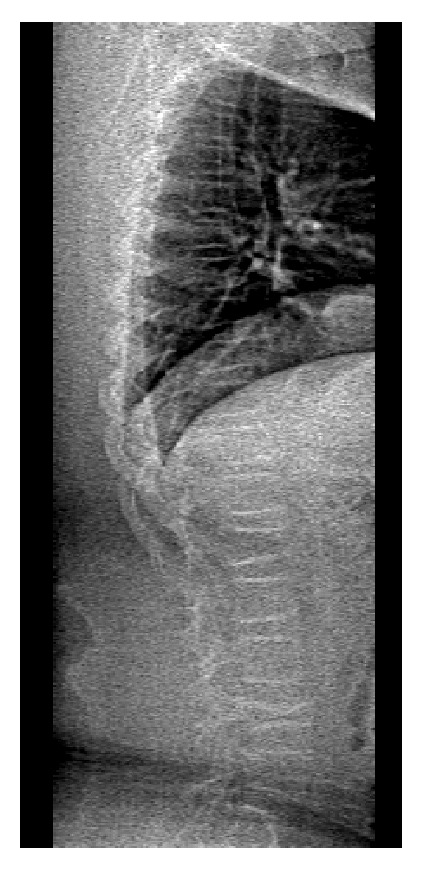
Densitometric lateral spinal imaging obtained at the time of bone density test by DXA shows L1 and L4 compression fracture with a mild anterior wedging of L2 in a 14-year-old boy with DMD. (Film courtesy of Dr. Sasigarn Bowden.)

Screening for vertebral fractures in corticosteroid-treated patients with DMD is essential, as prior vertebral fractures are a powerful predictor of subsequent fractures and, when present, are an indication for treatment with bisphosphonates. Spinal radiographs should be obtained when patients complain of back pain or height-adjusted lumbar spine *Z*-score is <−2 [29]. Separate films of the thoracic and lumbar spine are required to visualize the entire vertebral column at risk for fractures. Anterior wedging or biconcave deformity is indicative of a vertebral compression fracture. Currently, there is no consensus for when to perform spinal screening in asymptomatic patients. In our institution, we obtain a densitometric lateral spinal imaging (Figure 1) at the time of the DXA bone density test. This technique has been proven to be a low-cost method of vertebral fracture screening, which can be done efficiently and quickly at the time of DXA, with much less radiation exposure compared to a traditional spinal radiograph (10–20 *μ*Sv versus 800 *μ*Sv) [40]. This technique allows for vertebral screening in asymptomatic patients with DMD who undergo routine DXA BMD testing.

#### 5.2.2. BMD Measurement

Imaging methods for bone densitometry include dual energy X-ray absorptiometry (DXA) scan, quantitative computed tomography (QCT), and qualitative ultrasound; the DXA scan is the most widely used method due to its relatively low radiation exposure, precision, and opportunities to follow the skeleton sequentially over time. The current recommendation is to use (1) total body less head BMD and (2) lumbar spine BMD spine as the preferred sites of measurements because of the speed and precision of measurements and well-established pediatric normative data. The rationale for excluding the head in the total body bone density measurement is that the skull constitutes a large percentage of the total body bone mass but does not respond to growth, physical activity, or metabolic changes. Inclusion of the skull potentially under- or overestimates BMD gains or losses at other skeletal sites. Total body less head analysis of total body BMD has been shown to improve BMD assessment in boys with DMD [41]. Currently, there is not enough data to support routine BMD measurements of other skeletal sites, such as the forearm and proximal or distal femur; reference data for these sites are only available for certain machines and scan software as well as additional specialized personnel training would be required [41–43]. In children, DXA measurements of the hip region (total hip or femoral neck) are not as reliable due to difficulties in identifying the bony landmarks for this region of interest [43].

Baseline DXA scans should be obtained prior to starting glucocorticoids, every 1-2 years while on glucocorticoids and yearly if on bisphosphonate therapy [29]. DXA scans in children must be interpreted correctly; *T*-scores should not be used before 20 years of age, as this compares the patient's BMD with that of a healthy young adult. Age-adjusted *Z*-score is used in pediatric patients but this might not be appropriate in DMD patients with short stature, which would result in lower *Z*-score when compared to healthy children of normal height. More useful is the height-adjusted *Z*-score [43, 44] which represents the number of standard deviations; the BMD of a subject is away from the height-adjusted predicted mean BMD of that subject. This method corrects for the tendency for smaller children to have lower bone density than taller children of the same age. Additional factors that may affect BMD measurements include scoliosis, which may require placement of corrective hardware, and vertebral fractures. Both phenomena can lead to inaccurate spinal bone density measurement and an erroneous *Z*-score [44].

## 6. Treatments to Optimize Bone Health

### 6.1. Calcium and Vitamin D

Upon optimizing the calcium and vitamin D intake in patients with DMD, Bianchi et al. [45] reported that over 65% of 33 DMD patients on corticosteroid treatment displayed increased BMD after 12 months. Additional studies are needed to define the optimal vitamin D level for DMD patients; thus, current treatment goals are to maintain sufficient 25-hydroxyvitamin D levels [29]. Recommended daily calcium and vitamin D intake varies by age and is detailed in Table 2. Recommended adult vitamin D_3_ daily intake is 800–1,000 IU while the pediatric recommended daily intake is based on age [28]. Treatment recommendations for vitamin D insufficiency and deficiency are outlined in Table 1. Higher dose of vitamin D may be required in individuals with high adiposity, malabsorption, and dark skin pigmentation or those taking chronic glucocorticoids. Thus, it is important to monitor vitamin D levels periodically and increase the dosage if levels remain low.

### 6.2. Vibration Therapy

Whole body vibration has also been evaluated as a means to preserve or improve muscle and bone function in DMD patients. Patients tolerate whole body vibration well, but studies, even after 1 year of treatment, have not shown improvement in muscle function [46] or bone density [47]. A Cochrane review for whole body vibration in adults with Parkinson's disease and multiple sclerosis showed insufficient evidence to support beneficial effects [48]. However, in children with cerebral palsy, there have been modest improvements in bone mass with high-frequency and low-intensity vibrations seen at 6 months [49, 50] and 12 months of therapy [51]. More investigation is needed to determine whether longer duration of treatment or earlier intervention with vibration therapy would be beneficial for DMD patients.

### 6.3. Bisphosphonate Treatment

Bisphosphonate treatment has become increasingly used in pediatric patients with clinical bone fragility in the last 2 decades. Reports of bisphosphonate use in pediatric patients with DMD have been limited in the literature, typically with oral alendronate, intravenous pamidronate, or zoledronic acid (Table 3) [52–56]. The benefits of bisphosphonates include improvement in BMD and *Z*-scores [52, 55, 56] and amelioration of back pain after vertebral fracture [53, 55]. Interestingly, one study from Canada showed that bisphosphonate treatment improved survival [54]. Additional studies will be required to validate this finding. Overall, bisphosphonates were well tolerated in these studies [52–56]. Oral bisphosphonates carry the risk of esophagitis in those unable to sit up right for 30 minutes after taking the medication [57]; IV bisphosphonates may be a better option for DMD patients lacking truncal stability or inability to sit up reliably.

Many issues remain to be defined regarding the use of bisphosphonates in patients with DMD who exhibit clinical bone fragility and/or low bone density, as to the best dose, best preparation, ideal length of therapy, or whether patients should receive a drug holiday (time-off bisphosphonate therapy). Moreover, it is unclear whether low bone mass without fractures should justify prophylactic therapy with bisphosphonates, similar to what has been recommended in adults for prophylactic treatment of steroid-induced osteoporosis (prior to first fracture) [58]. Sarkozy et al. presented 43 DMD boys who received prophylactic oral risedronate for an average of 24 months whose median lumbar spine adjusted BMD* Z*-scores remained stable during treatment (0.11 at baseline and 0.21 at 3-year followup); however, 7 boys had a long bone fracture and 3 had vertebral fractures on therapy (the majority having been on glucocorticoids for >36 months by the time of fracture) [59]. More research is needed to address these important questions.

### 6.4. Prospective Treatments

Two possible treatments for low bone density that have not been evaluated in DMD patients are denosumab and recombinant parathyroid hormone (PTH). Denosumab is a human monoclonal antibody against receptor activator of nuclear factor-*κ*B-ligand (RANKL). Inhibiting RANKL, which leads to decreased osteoclast number and activity, results in less bone resorption [60]. Denosumab has been used in a pediatric patient with Paget's disease [60] and also a few children with osteogenesis imperfecta type VI [61]. Another possible treatment would be recombinant PTH (teriparatide). In a 19-year-old patient with osteoporosis pseudoglioma, teriparatide was used to increase BMD [62].

A new treatment (for DMD in general) under clinical study is ataluren, a molecule that binds with ribosomes and may allow the insertion of an amino acid in the premature termination codon, and exon-skipping, which binds with ribonucleic acid (RNA) and excludes specific sites of RNA splicing, producing a dystrophin that is a smaller but more functional molecule. There are also studies underway that attempt to modulate other muscular proteins, such as myostatin and utrophin, to reduce DMD symptoms [21].

Melatonin may also have a role in treating both muscle function and bone metabolism in DMD. Intraperitoneal injections and subcutaneous implants of melatonin demonstrated improved muscle function in the dystrophic mdx5Cv mouse model for DMD [63]. This improved function may be due to melatonin's ability to normalize proinflammatory cytokines (such as nitrites (NOx), interleukin- (IL-) 1*β*, IL-2, IL-6, tumor necrosis factor-*α*, and interferon-*γ*) and reduce oxidative stress [64]. When cultured with melatonin, mouse osteoblastic MC3T3-E1 cells showed increased differentiation and mineralization by Alizarin red S staining [65]. In* ddy* mice, pharmacological doses of melatonin demonstrated a 36% increase in BMD (*P* < 0.005) possibly by decreasing the RANKL (and thus reducing bone resorption) [66]. The muscle function and BMD benefits need to be investigated specifically in DMD patients prior to widespread use.

## 7. Conclusions

Poor bone health is a significant concern in DMD patients as it can contribute to loss of ambulation and adversely affect quality of life and possibly impact survival. There is a great need for evidence based practice guidelines of management of bone health in patients with DMD. Screening for vitamin D deficiency/insufficiency and low bone mass should be routinely pursued in all patients with DMD, particularly those treated with corticosteroids, while ensuring adequate vitamin D and calcium intake. More studies are needed to determine the timing of first bone density test and the clinical usefulness of biochemical bone turnover markers and other newer imaging techniques such as vertebral morphometry by DXA, peripheral quantitative computed tomography, and micromagnetic resonance imaging to accurately assess bone quality and predict fracture risk. Early intervention to improve bone health by vibration therapy requires more study to validate the benefits as seen in other neuromuscular diseases. Bisphosphonates have generally been recommended to treat bone fragility, but prospective randomized controlled studies with long-term followup are needed to confirm their antifracture efficacy and safety. New investigations as to the benefit of teriparatide, melatonin, or denosumab on BMD and fracture risk in this patient population should be explored.

## Figures and Tables

**Table 1:** Screening recommendations and course of action summary for children with DMD [26, 27].

Screening	Timing	Course of action
Back pain assessment	Each visit	If present, obtain vertebral imaging

Calcium intake and vitamin D intake (diet and amount of sun exposure)	Initial and subsequent visits	Calcium and vitamin D supplementation as needed; see Table 2.

Serum 25-hydroxyvitamin D	Every 1-2 years	Vitamin D insufficiency/deficiency treatment without clinical signs of rickets. Ergocalciferol or cholecalciferol dose based on vitamin D level: 20–30 ng/mL: 1000 IU PO daily, <20 ng/mL: 2000 IU PO daily, <10 ng/mL: 4000 IU PO daily. (i) Dose may need to be higher in patients with malabsorption, chronic glucocorticoids use, dark skin pigmentation, or obesity. (ii) Serum 25-OH vitamin D level should be repeated in 3 months after giving pharmacologic doses of vitamin D. (iii) When the level is optimal, vitamin D dose should be reduced to a supplementation dose at 400–800 IU/day (or higher in chronic glucocorticoid use).

Bone turnover markers	Not formally recommended at this time	Further research is needed and may be useful in monitoring bisphosphonate therapy.

DXA scan	Obtain baseline prior to glucocorticoid use every 1-2 years thereafter	If height-adjusted lumbar BMD *Z* score <−1, should repeat DXA in 1 year. Worsening BMD and/or BMD *Z* score or the gain in BMD is less than expected, consider vertebral imaging.

Vertebral imaging (X-rays or densitometric lateral spinal imaging)	Obtain if back pain present or lumbar height-adjusted *Z*-score < −2	If vertebral fracture is present, start bisphosphonate therapy.

**Table 2:** Recommended daily allowance for calcium and vitamin D [67].

Age	Calcium RDA^*∗*^ (mg/d)	Vitamin D RDA^*∗*^ (IU/D)
0–6 months old	200^*∗∗*^	400^*∗∗*^
6–12 months old	260^*∗∗*^	400^*∗∗*^
1–3 years old	700	600
4–8 years old	1000	600
9–13 years old	1300	600
14–18 years old	1300	600

^*∗*^RDA = recommended daily allowance.

^*∗∗*^RDAs not established, and thus values are adequate intake reference.

**Table 3:** Summary of bisphosphonate use in DMD patients.

Study	Year	Study type	Patient number	Steroids	Bisphosphonate	Mean Age at Bisphosphonate Initiation	Results	Comments
Houston et al. [52]	2014	Retrospective cohort	39	29 were on prednisone or deflazacort	Alendronate PO	12 years old (no range given)	*Z*-score trended up at the hip with alendronate, but it is not statistically significant	10 did not receive alendronate, varying dosages of alendronate used

Sbrocchi et al. [53]	2012	Retrospective observational	7	All but 1 were reported as prednisone equivalents	Pamidronate IV 9 mg/kg/year or zoledronic acid IV 0.1 mg/kg/year	11.6 years old (range: 8.5–14.3 years old)	Improved back pain and stabilization to improvement in vertebral height ratios for the previously fractured vertebrae	Only patients with vertebral fractures were included in the study

Gordon et al. [54]	2011	Retrospective observational	44	5 prednisone only; 13 changed from prednisone to deflazacort; 26 deflazacort only	11 used pamidronate only; 1 changed from pamidronate to alendronate; 3 alendronate only; 1 clodronate only	12.5 years old (range: 7–23 years old)	Survival curve showed improvement in survival rate (*P* = 0.005, log-rank test); also, possible therapy duration effect could be present (*P* = 0.007, log-rank test)	Pamidronate was IV; alendronate was PO; clodronate was PO

Atance et al. [55]	2011	Case reports	3	2 on deflazacort	Alendronate 10 mg daily PO	11.4 years old (range: 8.1–15.8 years old)	Reduced back pain and improved BMD	Only 3 patients were reported

Hawker et al. [56]	2005	Before-after trial	23	All on deflazacort	Alendronate 0.08 mg/kg/day PO	10.8 years old (range: 6.9–15.6 years old)	Positive effect on BMD and *Z*-scores, better BMD outcome when given early in the course of disease	Also received 750 mg daily calcium and 1000 IU vitamin D

## Abbreviations

DMD: Duchenne muscular dystrophy
CK: Creatine kinase
BMD: Bone mineral density
DXA: Dual energy X-ray bsorptiometry
OC: Osteocalcin
BALP: Bone-specific alkaline phosphatase
PINP: Procollagen intact amino-terminal propeptide
ICTP: Carboxyterminal telopeptide
NTx: N-terminal cross-linked telopeptide
TRACP-5b: Osteoclast-derived, tartrate-resistant, acid phosphatase isoform 5b
QCT: Quantitative computed tomography
PTH: Parathyroid hormone
RANKL: Receptor activator of nuclear factor *κ*B-ligand
RNA: Ribonucleic acid
RDA: Recommended daily allowance
IL: Interleukin.
